# Executive Function in Gambling Disorder: A Meta-analysis on Neuropsychological Evidence

**DOI:** 10.1007/s10899-025-10383-1

**Published:** 2025-04-10

**Authors:** Miguel Peixoto, Artemisa Dores, Maria Monteiro, António Marques, Fernando Barbosa

**Affiliations:** 1 Psychosocial Rehabilitation Laboratory, Center for Rehabilitation Research (LabRP-CIR), Escola Superior de Saúde (E2S), Instituto Politécnico Do Porto, 4200-072 Porto, Portugal; 2 Laboratory of Neuropsychophysiology, Faculty of Psychology and Education Sciences, University of Porto, 4200-135 Porto, Portugal

**Keywords:** Gambling disorder, Behavioral addiction, Executive function, Shifting, Inhibition, Verbal fluency, Working memory

## Abstract

Gambling disorder (GD) is associated with deficits in various cognitive functions. Specifically for executive function (EF), previous findings are inconsistent, despite deficits being reported for shifting, inhibition, planning, and working memory domains. Although a worse performance in EF measures related to GD severity is often reported, there is a need to clarify current evidence. This study aims to systematically review and perform a meta-analysis to clarify the association between EF deficits and GD. The current study followed the preferred reporting items for systematic reviews and meta-analyses protocols. The meta-analysis used a random effects model and robust variance estimation to analyze the data, using Hedge’s g to report effect sizes. A total of 21 studies were systematically reviewed, of which 17 were included for meta-analysis. Meta-analysis was performed for shifting, inhibition, planning, and verbal fluency. Due to a lower number of studies, working memory data was systematically reviewed, but no meta-analysis was performed. Significant effect sizes were found for shifting and inhibition, indicative of deficits in participants with GD. No significant deficits were found for the other EF domains. Working memory results revealed inconsistent evidence, even when divided into verbal and visuospatial modalities. There is enough evidence of deficits in shifting and inhibition in participants with GD. However, better sample characterization should be considered in future studies to better understand the sources of potential heterogeneity. Consideration of gambling severity as a continuous variable could allow for a more detailed analysis of EF alterations across the various degrees of GD.

## Introduction

Gambling disorder (GD) is included in the diagnostic and statistical manual of mental disorders-text revision (DSM-5-TR) in the “Substance-Related and Addictive Disorders” section, under the “Non-Substance Related Disorders” sub-section (American Psychiatric Association [APA], 2022). A total of 1.29% of the world population has GD, with 2.43% in a situation of moderate risk (Gabellini et al., 2022).

Individuals who participate in gambling activities can be classified into three groups based on their level of involvement: healthy/social gamblers (Joukhador et al., 2003; Temcheff et al., 2016); problematic gamblers (Joukhador et al., 2003) and pathological gamblers (Delfabbro, 2013; Shaffer & Korn, 2002). Healthy/social gamblers engage with gambling activities but do not fulfill the necessary criteria for being considered at-risk gamblers (Temcheff et al., 2016). According to the literature, this group tends to not lie about their involvement in gambling activities and has fewer distorted cognitions about gambling (e.g., illusion of control) (Joukhador et al., 2003; Temcheff et al., 2016). Problematic and pathological gamblers can be considered part of a continuum and seem to differ in their cognitive functioning, with pathological gamblers having the more severe level of involvement and exhibiting cognitive or/and behavioral dysfunctions that meet the criteria for a psychiatric disorder (Delfabbro, 2013).

Given these apparent deficits, several studies have investigated gamblers’ cognitive functioning, with increased impulsivity (Grant et al., 2016; Ioannidis et al., 2019) and insensitivity to loss (Genauck et al., 2017; Van Holst et al., 2010), being frequently reported. Changes in reward and punishment processing seem to be reflected in gamblers’ neurocognitive profile, with alterations in the mesolimbic pathway associated with GD (Goudriaan & Van Holst, 2012). These alterations are thought to affect the decision-making process in these individuals, characterized by both a tendency to make riskier decisions (Conversano et al., 2012; Goudriaan & Van Holst, 2012) and maintaining the same decision pattern even when the choices are disadvantageous (Conversano et al., 2012; Kapsomenakis et al., 2018). Decision-making may be influenced by several individual variables (Mestre-Bach et al., 2020a); for example, older adults seem to make more disadvantageous choices (Fein et al., 2007), but, in certain tasks, this association is moderated by logical reasoning and cool cognitive functions, namely working memory (Colautti et al., 2022).

Potentially underlying a behavior characterized by high impulsivity and riskier decision-making, are executive function (EF) deficits (Mallorquí-Bagué et al., 2018; Reynolds et al., 2019). Considering, on the one hand, that EF can be defined as the ability to plan, organize, adjust and carry out complex goal-oriented tasks (Oscar-Berman et al., 2014), and its correlation with addictive behaviors on the other (Kräplin et al., 2022), the study of EF in individuals with GD could foster our understanding of the pathology. Indeed, existing literature suggests various EF components to be affected in this population, although an overall executive deficit is not consensual (Kapsomenakis et al., 2018).

Analyzing each EF domain also reveals a lack of consensus. Shifting/cognitive flexibility can be defined as the ability to switch between multiple tasks, or mental states effectively (Miyake et al., 2000). The data is inconsistent in determining the presence of shifting deficits in GD, with some studies pointing to a worse performance (Leppink et al., 2016a) and others showing no difference between GD samples and controls (Conversano et al., 2012). There is also evidence of reduced shifting after losses (Van Holst et al., 2010), potentially corroborating reduced cognitive flexibility in participants with GD. Inhibition is also affected in individuals with GD (Conversano et al., 2012; Kapsomenakis et al., 2018). This domain may be characterized as the ability to inhibit a more dominant or automatic response (Miyake et al., 2000). In the context of GD, it has been reported that impaired inhibition results in more impulsive behavior (Kapsomenakis et al., 2018), and is associated with greater delay discounting (Goudriaan & Van Holst, 2012; Van Holst et al., 2010). For planning, the ability to establish and sequence subgoals towards an overall goal (Hudson & Farran, 2011), participants with GD show a worse performance compared to healthy controls (HC) (Goudriaan et al., 2006), with worse results in more demanding tasks (Conversano et al., 2012). Verbal fluency is also considered a suitable indicator of EF (Shao et al., 2014) as it facilitates information retrieval from memory while requiring executive control over the necessary functions to do so (Patterson, 2011). In GD research, studies are inconsistent in determining whether gamblers show an impaired performance in this domain (Conversano et al., 2012). Working memory can be described as the capacity to temporarily hold information in a heightened state of availability for use in an ongoing process (Cowan, 2017). A verbal modality, capable of storing phonological information and articulatory rehearsal, and a visuospatial modality, capable of storing and manipulating visual and spatial information, can be considered (RepovŠ & Baddeley, 2006). This EF domain is potentially altered in GD, negatively correlating with gambling severity (Ngetich et al., 2023), perseveration errors (Van Holst et al., 2010), and decision-making (Ngetich et al., 2023). However, evidence is contradictory, with studies reporting GD samples with similar (Ngetich et al., 2023) or even better performance than HC (Kapsomenakis et al., 2018) in working memory tasks. Tied to working memory is updating, an EF domain responsible for manipulating the information in working memory, assuring the stored information remains relevant (Miyake et al., 2000). Despite being closely linked, evidence suggests that processes such as maintaining information in working memory are associated with brain structures (e.g. medial prefrontal cortex; mPFC) that are not involved in updating (Trutti et al., 2021). Furthermore, while simpler span or delay recognition-tasks are more impervious to the effects of updating, N-back tasks require this EF (D’Esposito & Postle, 2002). In spite of these differences, few studies in this field disentangle working memory from updating (Ngetich et al., 2023): as such, specific impairments in this domain are harder to ascertain.

Bearing in mind the current inconsistencies in evidence regarding the link EF-GD, it is important to systematically review and meta-analyze such evidence. The study of Conversano et al. (2012) provided a review of the data gathered about EF alterations between 1995 and 2011. Our study aims to update their results, while performing meta-analysis to more accurately characterize EF in individuals with GD. A more recent study (Van Timmeren et al., 2018) performed a meta-analysis focused on compulsivity-related neurocognitive functioning, which included the analysis of some EF domains. Our study will extend these findings with a broader analysis of EF domains. Based on current data, we aim to perform a systematic review and meta-analysis to analyze current evidence on executive dysfunctions in samples with GD, using standardized neuropsychological instruments that assess the variables of interest. In addition, we also aim to analyze the instruments that are more capable of distinguishing individuals with GD from HC, in terms of their EF.

Based on current evidence we formulated the hypotheses that shifting, inhibition, and planning are significantly impaired in participants with GD, while verbal fluency and working memory are not. For updating, we do not expect enough studies to perform a quantitative review.

## Methods

The following search string was formulated based on the literature to collect the necessary data: (“gambl* disorder” OR “pathological gambl*” OR “problematic gambl*”) AND (“executive function*” OR “executive dysfunction” OR “dysexecutive syndrome”) NOT (“validat*” OR “animal*” OR “parkinson” OR “drug*” OR “substance” OR “intervention”). The search string was used in the Web of Science, Scopus, and EBSCOhost databases. In Web of Science and EBSCOhost the search was done within the abstract. In Scopus the search was done within the title, abstract, and keywords. A filter for publication year (2012–June 2024) was used across databases. An additional search was performed on PubMed, with the following search string: (“pathological gambling”[MeSH] OR “gambling disorder” OR “pathological gambling” OR “problematic gambling”) AND (“Executive Function”[MeSH] OR “executive function” OR “executive dysfunction” OR “dysexecutive syndrome”) NOT (“validation” OR “animal”). A filter for publication year (2012–January 2025) was used.

To gather studies of interest, the inclusion criteria were: (a) Adult participants—18 years or older; (b) Participants with GD and healthy controls/social gamblers; (c) Use of standardized neuropsychological instruments to measure EF; (d) Articles published in English or Portuguese in peer-reviewed journals; and (e) Articles published after 2012. Additionally, exclusion criteria were: (a) Main diagnosis of substance use disorder; (b) Main diagnosis of other psychopathologies: ADHD, bipolar disorder, obsessive–compulsive disorder, or schizophrenia; (c) Main diagnosis of traumatic brain injury or other neurological disorders; (d) Neuroimaging studies; (e) Studies outside of the review scope; (f) Systematic reviews; (g) Meta-analyses; (h) Case studies; (i) Conference presentations and conference books; and (j) Book chapters.

The review was performed according to the Preferred Reporting Items for Systematic Reviews and Meta-Analyses statement (PRISMA; Page et al., 2021) and Cochrane collaboration guidelines (Higgins & Green, 2019). After data collection, two reviewers (M.P.; M.M.), working independently, carefully screened the title and abstracts of the extracted studies against the defined inclusion and exclusion criteria. This process was done through the Rayyan software (Ouzzani et al., 2016). At the end of the selection process, the reviewers met to converge their results, with any disagreements resolved by a third expert contributor (A.R.D.). Afterwards, the full text of the selected studies was analyzed by one reviewer (M.P.). To prevent publication and source selection bias, an additional hand search was performed based on the reference list of selected studies. The same procedure was performed for PubMed, with the selection process being carried out independently by two reviewers (M.P.; M.F.).

Two independent reviewers assessed the quality of the included studies using the Appraisal Tool for Cross-Sectional Studies (AXIS), a tool developed to assess the quality and risk of bias of cross-sectional studies. AXIS is comprised of 20 items regarding the introduction, methods, results, and discussion of each study. Cohen’s Kappa was used to assess interrater agreement, with disagreements being solved by consensus (Downes et al., 2016).

Data extracted from each study included: (a) Study information—Authors and publication year; (b) Sample characteristics—Sample size, gender ratio, age, formal education level, treatment status, comorbidities, gambling severity; (c) Variables assessed—EF domain assessed and other cognitive functions; (d) Assessment protocol—Instruments used and their related measures; and (e) Results related to EF—Group comparisons and/or correlations with gambling severity.

For the meta-analysis, neuropsychological measures were organized per EF domain following Snyder et al. (2015). For a specific EF domain to be included in the analysis, it had to be assessed in at least three studies. For effect size calculation in studies conducting between-groups comparisons, means and standard deviations for each group (GD and HC) were collected. Alternatively, Cohen’s *d* and exact *p*-values were used. Correlations were also included, provided the correlation coefficients were computed between EF measures and measures of gambling severity, such as the scores of the South Oaks Gambling Screen (SOGS), or the Problem Gambling Severity Index (PGSI). Effect sizes were always computed so that a positive effect reflected better performance by HC samples.

The corresponding authors of the included studies were contacted via e-mail whenever the necessary data for calculating effect sizes was not reported in the study. If the data was not provided by the authors, the study entry was excluded from meta-analysis. A total of six authors were contacted for missing information. One author answered back.

Meta-analysis procedures were conducted using the ProMeta 3.0 software (Internovi, 2015), and the “metaphor” (Viechtbauer, 2010) and “clubSandwich” (Pustejovsky, 2024) packages for R (R core team, 2024). Given that some studies reported multiple instruments assessing the same EF domain, robust variance estimation was used to account for dependent effect sizes (i.e., effect sizes derived from the same sample) (Pustejovsky & Tipton, 2022). A random effects model was used for all analyses. Effect sizes were calculated as Hedges’ *g* (Hedges, 1981), as this index is more adjusted for studies with smaller sample sizes. Due to the variety of EF measures, they were coded into three categories: positive (if a higher score was indicative of a better performance), negative (if a higher score was indicative of a worse performance), and response time (RT; if time was used as a measure of performance). With studies reporting multiple measures sharing the same category (e.g., a study reports commission and omission errors, both measures coded as negative, to assess the performance on the Continuous Performance Task; CPT), measure selection was based on the study of Snyder et al. (2015). For instruments reported in three or more studies, an overall effects model was assessed using the related measures. The overall effects model for each EF was calculated using the instrument-related measures. A moderator analysis was performed with the variables of interest being age, gender, and years of formal education, per group (GD and HC). Due to the low number of studies assessing gambling severity as a continuous variable, moderator analysis was not possible.

Heterogeneity in the overall effect models was assessed using the *Q* statistic (Cochran, 1954), with its statistical significance indicating a significant heterogeneity. The *Q* statistic results were complemented by the *I*^2^ statistic and prediction intervals. Prediction intervals were estimated using effect size, T^2^ values and the Z-value of 1.96, which corresponds to the confidence level of 95% (Borenstein et al., 2009). A study was deemed an outlier based on its standardized residual (> 3; Viechtbauer & Cheung, 2010). Entries identified as outliers were removed and effect size and heterogeneity were computed again to verify if removing the study increased precision, based on significance of the overall model and heterogeneity. Publication bias was assessed through the trim-and-fill method (Shi & Lin, 2019), complemented by Egger’s test (Egger et al., 1997).

## Results

**Fig. 1:**
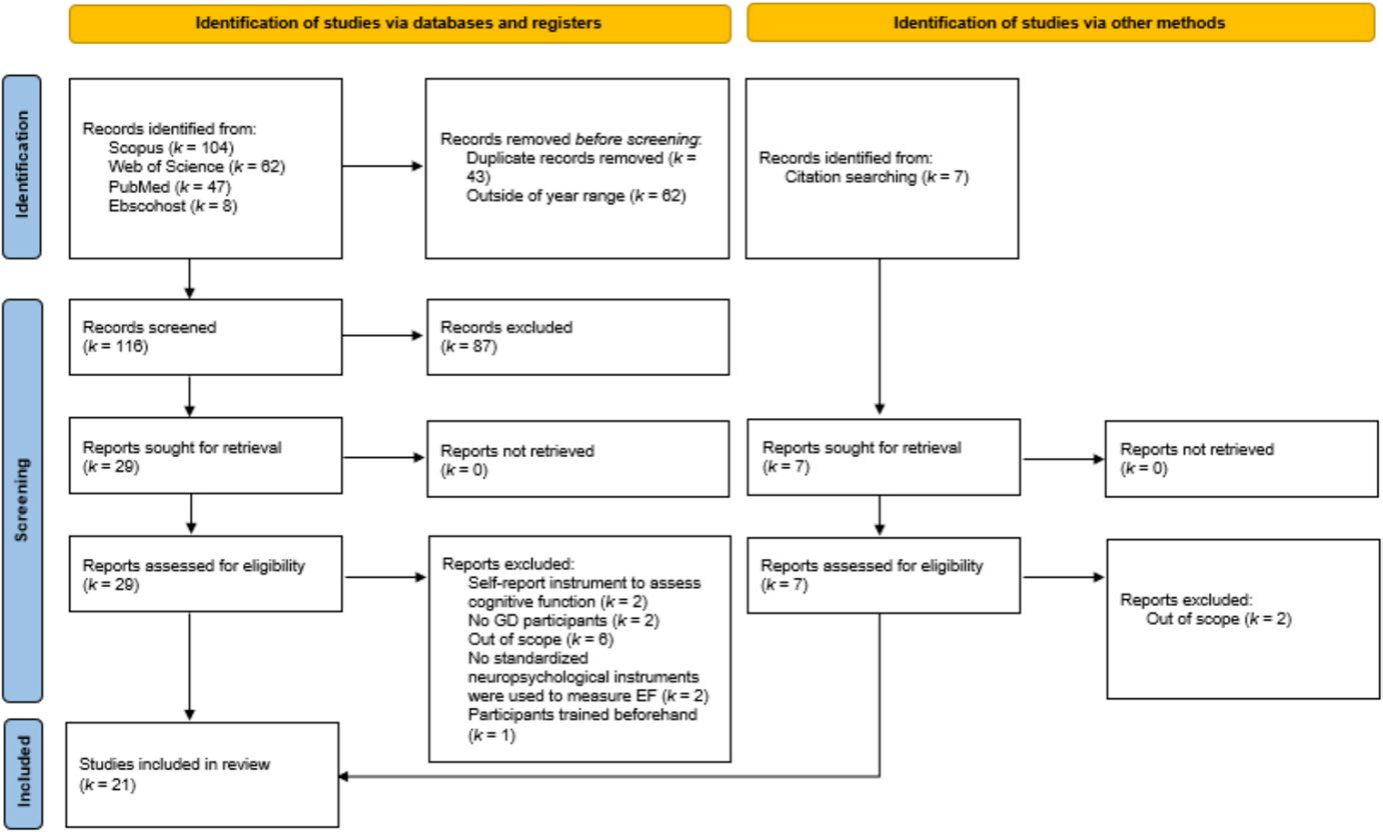
PRISMA 2020 flow diagram for systematic reviews

A total of 221 studies were identified following the systematic search of the databases. Figure 1 shows the PRISMA flow diagram with the study selection process. Before screening, duplicate records (*k* = 43) and records published before 2012 (*k* = 62) were removed. The title and abstract of the remaining 116 articles were screened by reviewers, with 87 being excluded. Thus, 29 were retrieved for a full-text analysis. Cohen’s kappa was used to assess interrater agreement between M.P. and M.M., with a score of 0.60, classified as a moderate agreement (McHugh, 2012). For the PubMed search, the interrater agreement was 0.62 classified as a moderate agreement (McHugh, 2012).

Based on the full-text analysis, 13 articles were excluded for the following reasons: (a) Use of self-report instruments to assess cognitive function (*k* = 2); (b) No participants with GD (*k* = 2); (c) Out of scope (*k* = 6); (d) No standardized neuropsychological instruments were used to measure EF (*k* = 2); and (e) Other reasons, namely participants were trained on a strategy to enhance task performance beforehand (*k* = 1). To avoid search bias, an additional hand search was performed, leading to the identification and inclusion of five additional studies. Thus, a total of 21 studies were included in the systematic review.

For quality and risk assessment, Cohen’s kappa was used to assess interrater agreement, with a score of 0.86, classified as strong agreement (McHugh, 2012). Common weaknesses were observed across the majority of studies. A prominent issue was the lack of sample size justification, with most studies not reporting a clear rationale for sample size. This could potentially limit the statistical power of their results, thereby affecting the validity of the conclusions drawn. The lack of data provided on non-responders was another frequent issue. Non-responders could be part of a specific group, and their exclusion could limit the applicability of results to that group. Despite these limitations, quality and risk assessment revealed an overall compliance with the standards established by AXIS (Table 1), reflecting high quality standards.

**Table 1:** Appraisal tool for cross-sectional studies (AXIS)

Studies																					
Axis	1	2	3	4	5	6	7	8	9	10	11	12	13	14	15	16	17	18	19	20	21
*Introduction*																					
1	Yes	Yes	Yes	Yes	Yes	Yes	Yes	Yes	Yes	Yes	Yes	Yes	Yes	Yes	Yes	Yes	Yes	Yes	Yes	Yes	Yes
Methods																					
2	Yes	Yes	Yes	Yes	Yes	Yes	Yes	Yes	Yes	Yes	Yes	Yes	Yes	Yes	Yes	Yes	Yes	Yes	Yes	Yes	Yes
3	No	No	No	Yes	No	No	No	No	No	No	No	No	No	No	No	No	No	No	Yes	No	No
4	Yes	Yes	Yes	Yes	Yes	Yes	Yes	Yes	Yes	Yes	Yes	Yes	Yes	Yes	Yes	Yes	Yes	Yes	Yes	Yes	Yes
5	Yes	Yes	Yes	Yes	Yes	Yes	Yes	Yes	Yes	No	Yes	No	Yes	Yes	Yes	Yes	Yes	Yes	Yes	Yes	Yes
6	Yes	Yes	Yes	Yes	Yes	Yes	Yes	Yes	Yes	No	Yes	Yes	No	Yes	Yes	No	Yes	Yes	Yes	Yes	Yes
7	Yes	No	No	No	No	No	Yes	No	No	No	No	No	No	Yes	No	Yes	No	Yes	No	No	No
8	Yes	Yes	Yes	Yes	Yes	Yes	Yes	Yes	Yes	Yes	Yes	Yes	Yes	Yes	Yes	Yes	Yes	Yes	Yes	Yes	Yes
9	Yes	Yes	Yes	Yes	Yes	Yes	Yes	Yes	Yes	Yes	Yes	Yes	Yes	Yes	Yes	Yes	Yes	Yes	Yes	Yes	Yes
10	Yes	Yes	Yes	Yes	Yes	Yes	Yes	Yes	Yes	Yes	Yes	Yes	Yes	Yes	Yes	Yes	Yes	Yes	Yes	Yes	Yes
11	Yes	Yes	Yes	Yes	Yes	Yes	Yes	Yes	Yes	Yes	Yes	Yes	Yes	Yes	Yes	Yes	Yes	Yes	Yes	Yes	Yes
*Results*																					
12	Yes	Yes	Yes	Yes	Yes	Yes	Yes	Yes	Yes	No	Yes	No	Yes	Yes	Yes	No	Yes	Yes	Yes	Yes	Yes
13	No	No	No	No	No	No	No	No	No	Yes	No	Yes	No	No	No	No	No	No	No	No	No
14	Yes	Yes	Yes	Yes	Yes	Yes	No	No	Yes	No	Yes	No	Yes	Yes	Yes	No	Yes	Yes	Yes	Yes	No
15	Yes	Yes	Yes	Yes	Yes	Yes	Yes	Yes	Yes	Yes	Yes	Yes	Yes	Yes	Yes	Yes	Yes	Yes	Yes	Yes	Yes
16	Yes	Yes	Yes	Yes	Yes	Yes	Yes	Yes	Yes	Yes	Yes	Yes	Yes	Yes	Yes	Yes	Yes	Yes	Yes	Yes	Yes
*Discussion*																					
17	Yes	Yes	Yes	Yes	Yes	Yes	Yes	Yes	Yes	Yes	Yes	Yes	Yes	Yes	Yes	Yes	Yes	Yes	Yes	Yes	Yes
18	Yes	Yes	Yes	Yes	Yes	Yes	Yes	Yes	Yes	Yes	Yes	Yes	Yes	Yes	Yes	Yes	Yes	Yes	Yes	Yes	Yes
*Othe*r																					
19	No	No	DN	No	No	No	Yes	No	No	No	No	No	No	No	No	DN	No	No	No	No	No
20	No	Yes	Yes	Yes	Yes	Yes	Yes	Yes	Yes	Yes	Yes	Yes	Yes	Yes	Yes	Yes	Yes	Yes	Yes	Yes	Yes

DN, don’t know

Sample characteristics are presented in Table 2. The included studies comprised 859 participants who fulfilled the criteria for GD. A total of 498 participants were identified as male and 152 as female. Two studies did not report sex (Albein-Urios et al., 2012; Kertzman et al., 2018), and another did not report the sex of the sub-sample that underwent neuropsychological assessment (Medeiros et al., 2017). Two studies involved 552 participants with varying degrees of gambling severity (Leppink et al., 2016a; Schiavella et al., 2018). On average, participants with GD were 40 years old (*SD* = 5.70), with one study (Medeiros et al., 2017) not reporting the age of the participants that completed the neuropsychological instruments. Most studies (*k* = 13) recruited samples with individuals undergoing treatment for GD, with others recruiting participants from the community (*k* = 6), or from both sources (*k* = 2).

**Table 2:** Sample characteristics of the included studies

Studies	Country	Participant Type (*n*)	Sex (*n*)	Age *M (SD)*	Educational Level *M (SD)*	Treatment Status (*n*)	Co-morbidities
Albein-Urios et al., (2012)	Spain	GD: (23) HC: (20)	Not reported	GD: 35.6 (8.7) HC: 28.6 (3.6)	GD: 9.8 (2.2) HC: 10.6 (1.8)	In treatment (23)	Reported: No history of psychiatric disorder; No history of neurological disorder; No history of TBI Not reported: History of substance abuse
Ledgerwood et al., (2012)	Canada	GD: (45) HC: (45)	GD: M (24) F (21) HC: M (22) F (23)	GD: 46.1 (13.9) HC: 45.8 (17.3)	GD: 14.2 (2.5) HC: 14.4 (2.5)	In treatment (23) Community (22)	Reported: No history of substance abuse; No history of psychiatric disorder Not reported: History of TBI; History of neuropsychological disorder
Hur et al., (2012)	Korea	GD: (16) HC: (52)	GD: M (16) HC: M (36) F (16)	GD: 28.31 (3.79) HC: 25.13 (5.00)	GD: 14.88 (1.67) HC: 14.35 (1.53)	In treatment (16)	Reported: No history of substance abuse disorder; No history of TBI; No history of psychiatric disorder; No history of neurological disorder
Manning et al., (2013)	Singapore	GD: (30) Skill gamblers (25) Luck gamblers (5) HC: (30)	GD: M (30) HC: M (30)	GD: 37.1 (8.9) HC: 37.2 (11.7)	GD: 12.4 (2.0) HC: 12 (2.4)	In treatment (30)	Reported: Suicidal ideation in 11 GD: History of psychiatric disorder in 7 GD; No history of neurological disorder; No history of TBI
Boog et al., (2014)	Netherlands	GD: (19) HC: (19)	GD: M (14) F (5) HC: M (16) F (3)	GD: 42.1 (13.35) HC: 38.8 (8.0)	GD: 13.47 (4.0) HC: 15.11 (2.47)	In treatment (19)	Reported: No history of psychiatric disorder; No history of neurological disorder Not reported: History of substance abuse; History of TBI
Choi et al., (2014)	Korea	GD: (15) HC: (15)	GD: M (15) HC: M (15)	GD: 27.53 (5.21) HC: 25.33 (5.30)	GD: 14.73 (2.63) HC: 14.53 (1.85)	In treatment (15)	Reported: No history of substance abuse Not reported: History of psychiatric disorder; History of neurological disorder; History of TBI
Kräplin et al., (2014)	Netherlands	GD: (51) HC: (53)	GD: M (44) F (7) HC: M (32) F (21)	GD: 37.82 (9.97) HC: 36.74 (11.41)	Not reported	In treatment (51)	Reported: History of substance abuse; History of psychiatric disorder Not reported: History of TBI
Yan et al., (2014)	China	GD: (58) HC: (60)	GD: M (58) HC: M (60)	GD: 35.6 (7.1) HC: 34.3 (8.5)	GD: 8.9 (3.2) HC: 9.5 (2.6)	In treatment (58)	Reported: No history of substance abuse; No history of psychiatric disorder; No history of neurological disorder; No history of TBI
Leppink et al., (2016a)	U.S.A	GD & HC: (552)	GD & HC: M (361) F (191)	GD & HC: 22.20 (3.57)	GD & HC: College or higher education (n = 491)	In treatment (552)	Reported: No history of substance abuse Not reported: History of psychiatric disorder; History of neurological disorder; History of TBI
Leppink et al., (2016b)	U.S.A	GD: (101) (Completed BIS) GD: (80) (Completed SST) GD: (106) (Completed EIQ)	M (44) F (57) (Completed BIS) M (43) F (37) (Completed SST) M (50) F (56) (Completed EIQ)	45.93 (10.66) ^a^ (Completed BIS) 47.1 (12.28) (Completed SST) 45.83 (10.82) (Completed EIQ)	High school diploma or higher (57) (Completed BIS) High school diploma or higher (34) (Completed SST) High school diploma or higher (55) (Completed EIQ)	In treatment (80)	Reported: No history of substance abuse; No history of psychiatric disorder Not reported: History of neurological disorder; History of TBI
Zhou et al., (2016)	China	GD: (23) HC: (23)	GD: M (18) F (5) HC: M (16) F (7)	GD: 29 (6) HC: 28 (6)	GD: 9 (3) HC: 9 (3)	In treatment (23)	Reported: No history of substance abuse; No history of neurological disorder; No history of TBI
Medeiros et al., (2017)	U.S.A	GD: (448—> 77)^b^	GD: M (197) F (251)	47.6 (11.3)	Less than college (164) College or more (283)	Community (448)	Reported: No unstable medical illness; No psychotic symptoms; Affective disorder (27.2%); Alcohol use (23.2%); Substance abuse (11.7%)
Ellis et al., (2018)	Canada	GD: (38) HC: (40)	GD (depression or dysthymia): M (8) F (11) GD (no depression or dysthymia): M (11) F (8) HC: M (21) F (19)	GD (depression or dysthymia): 49.11 (12.02) GD (no depression or dysthymia): 43.63 (16.42) HC: 46.10 (17.42)	GD (depression or dysthymia): 14.6 (2.57) GD (no depression or dysthymia): 14.68 (2.45) HC: 14.44 (2.60)	In treatment Community	Reported: No history of substance abuse; No history of psychiatric disorder Not reported: History of TBI; History of neuropsychological disorder
Kapsomenakis et al., (2018	Greece	GD: (24) HC: (21)	GD: M (24) HC: M (21)	GD: 45.5 (10.6) HC: 44.7 (11.8)	GD: 14.58 (4.17) HC: 14.9 (3.95)	Community (24)	Reported: No history of psychiatric disorder; No history of neurological disorder; No history of TBI; 10 GD with history of substance abuse
Kertzman et al., (2018)	Israel	GD: (109) HC: (131)	Not reported	GD: 38.76 (13.11) HC: 35.67 (11.62)	GD: 13.46 (2.86) HC: 14.82 (3.08)	In treatment (109)	Reported: No history psychiatric disorder; No history of neurological disorder; No history of substance abuse Not reported: History of TBI
Schiavella et al., (2018)	Italy	Stage 1: HC & GD: (46) Stage 2: ^c^ HC & GD: (36)	Stage 1: M (41) F (5) Stage 2: M (31) F (5)	Stage 1: 32 (7.1) Stage 2: 33 (7.3)	15 (3)	Stage 1: Community (46) Stage 2: Community (36)	Not reported: History of psychiatric disorder; History of neurological disorder; History of TBI; History of substance abuse
Sharif-Razi et al., (2019)	Canada	GD: (27) HC: (21)	GD: M (17) F (10) HC: M (11) F (10)	GD: 45 (15.31) HC: 46.19 (13.33)	GD: 14.44 (2.12) HC: 16.05 (2.01)	Community (27)	Reported: No history of psychiatric disorder; No history of neurological disorder; History of alcohol consumption Not reported: History of TBI
Mestre-Bach et al., (2020b)	Spain	GD: (97) HC: (32)	GD: M (97) HC: M (32)	GD: 35.0 (8.8) HC: 31.3 (6.6)	GD: Primary school: 55.7%; Secondary school: 40.2%; University: 4.1% HC: Primary school: 9.4%; Secondary school:53.1%; University: 37.5%	In treatment (97)	Reported: 8 GD with substance abuse history; No history of psychiatric disorder Not reported: History of neurological disorder; History of TBI
Penolazzi et al., (2020)	Italy	GD: (30) HC: (30)	GD: M (21) F (9) HC: M (18) F (12)	GD: 49.27 (13.65) HC: 50.33 (13.52)	GD: 10.50 (3.30) HC: 12.23 (4.91)	In treatment (30)	Reported: No history of neurological disorder; 30% of participants with history of substance abuse; 13.3% of participants with history of psychiatric disorder Not reported: History of TBI
Mallorquí-Bagué et al., (2021)	Spain	GD: (57) Luck gamblers (46) Skill gamblers (11) HC: (60)	GD: M (30) F (27) HC: M (30) F (30)	GD: 45.7 (10.1) HC: 37.7 (11.6)	GD: 10.5 (3.4) HC: 14.3 (3.9)	Community (57)	Reported: No history of neurological disorder; No history of TBI Not Reported: History of psychiatric disorder; History of substance abuse
Aidelbaum et al., (2023)	Canada	GD: (40) HC: (50)	GD: M (28) F (12) HC: M (18) F (31)	GD: 44 (15.32) HC: 41.76 (13.83)	GD: 14.1 (1.85) HC: 15.82 (1.67)	Community (40)	Reported: Bipolar disorder: 5% Substance abuse disorder: 55% Obsessive compulsive disorder: 2.5% Trauma related disorder: 5% ADHD: 5% No reported: History of neurological disorder; History of TBI

GD, gambling disorder; HC, healthy control; TBI, traumatic brain injury; a, calculated weighted means and pooled standard deviations; b, Only 77 did neuropsychological assessment; c, Only stage 2 did neuropsychological assessment

A total of six EF domains are analyzed in the current review, specifically shifting (*k* = 12), inhibition (*k* = 17), planning (*k* = 6), verbal fluency (*k* = 5), working memory (*k* = 10), and updating (*k* = 1). Table 3 provides instruments and related measures for assessing EF domains, along with their results. Brief information on instruments used to assess other cognitive functions is also reported. Given the lack of studies that specifically address updating this domain cannot be further analyzed.

**Table 3:** Instruments characteristics, and executive function results of the included studies

Studies	Participant Type (*n*)	Instruments—Gambling	Instruments—Cognitive Function (Other)	Instruments—Cognitive Function (Executive Function)
Albein-Urios et al., (2012)	GD: (23) HC: (20)	SCID	Impulsivity: UPPS-P Delay discounting: Delay-discounting questionnaire IQ: Kaufman Brief Intelligence Test Personality: IPED	Inhibition: STROOP Updating: N-back
Ledgerwood et al., (2012)	GD: (45) HC: (45)	CPGI SCID NODS-lifetime NODS-Past year	Intelligent Quotient (IQ): WASI Decision-making: IGT	Shifting: WCST Inhibition: STROOP; Go/No-Go Planning: TOL Verbal fluency: COWAT Working memory: WMS
Hur et al., (2012)	GD: (16) HC: (52)	SOGS DSM-IV	IQ: WAIS Verbal memory: CVLT Visual memory: ROCF	Shifting: WCST; TMT Inhibition: STROOP Verbal fluency: COWAT; CFT
Manning et al., (2013)	GD: (30) Skill gamblers (25) Luck gamblers (5) HC: (30)	SOGS PGSI DSM-IV-TR	Fluid intelligence: RPM Impulsivity: BIS Cognitive lapses: CFQ Dysexecutive syndrome, emotional, and behavioural issues: DEX Decision-making: IST	Shifting: IED Planning: SOC Working memory (visuospatial): SWMT
Boog et al., (2014)	GD: (19) HC: (19)	SOGS	Psychological symptoms: BSI Obsessive–compulsive disorder: PI-R Cognitive flexibility (Decision-making): PRLT	Shifting: WCST
Choi et al., (2014)	GD: (15) HC: (15)	PGSI DSM-V	Impulsivity: BIS	Shifting: TMT; IED Inhibition: SST
Kräplin et al., (2014)	GD: (51) HC: (53)	DSM-IV	Impulsivity: BIS; Custom gambling task Decision-Making: IGT	Inhibition: SST; STROOP Planning: TOL
Yan et al., (2014)	GD: (58) HC: (60)	SOGS SCI-PG	Decision-Making: IGT	Working memory: SOPT
Leppink et al., (2016a)	GD & HC: (552)	SCI-PG	Impulsivity: BIS	Shifting: IED
Leppink et al., (2016b)	GD: (101) (Completed BIS) GD: (80) (Completed SST) GD: (106) (Completed EIQ)	SCID GSAS DSM-IV CGI	Impulsivity: BIS; EIQ	Inhibition: SST
Zhou et al., (2016)	GD: (23) HC: (23)	DSM-IV	Impulsivity: BIS	Shifting: WCST Inhibition: Go/No-Go Working memory (verbal): DST
Medeiros et al., (2017	GD: (448—> 77)	G-SAS DSM-IV	-	Shifting: IED Inhibition: SST
Ellis et al., (2018)^a^	GD: (38) HC: (40)	NODS SCID	IQ: WASI Impulsivity: BIS	Shifting: WCST Inhibition: STROOP Planning: TOL Working Memory: WMS Verbal Fluency: COWAT
Kapsomenakis et al., (2018)	GD: (24) HC: (21)	SOGS DSM-V	Memory: MIS Decision-making: IGT Auditory comprehension: CIG Processing speed: SDMT	Shifting: TMT Inhibition: STROOP Working memory (verbal): DST Working memory (visuospatial): CBTT Verbal fluency: COWF
Kertzman et al., (2018)	GD: (109) HC: (131)	SOGS	-	Inhibition: Go/No-Go; CPT
Schiavella et al., (2018)	Stage 1: GD (46) Stage 2: ^b^ GD (36)	SOGS PGSI GRCS	Fluid Intelligence: RPM Sustained and selective attention: MAT Emotional-social intelligence: Eqi:S Overwriting impulsive answer: CRT Risk-seeking: RSBQ Delay discounting: IBQ Dysexecutive syndrome: FAB Estimation: COGST Visuoespatial, planning, and praxic: FDCT	Shifting: WCST; TMT Inhibition: STROOP Planning: TOL Working memory (verbal): DST Working memory: MEMINT Verbal fluency: FLUS; FLUF
Sharif-Razi et al., (2019)	GD: (27) HC: (21)	PGSI CIDI	Impulsivity: UPPS-P	Inhibition: SSAT
Mestre-Bach et al., (2020b)	GD: (97) HC: (32)	SOGS DSM-V	Impulsivity: UPPS-P Delay discounting: Custom Task	Inhibition: CPT
Penolazzi et al., (2020)	GD: (30) HC: (30)	SOGS GABS GFA GRCS DSM-IV-TR interview	Impulsivity: BIS-11 Memory: RPP	Inhibition: Go/No-Go
Mallorquí-Bagué et al., (2021)	GD: (57) Luck gamblers (46) Skill gamblers (11) HC: (60)	DSM-V	-	Shifting: WCST; TMT Inhibition: STROOP
Aidelbaum et al., (2023)	GD: (40) HC: (50)	PGSI DSM-V	Risk taking: BART Delay discounting: DDT	Inhibition: CWIT; SSAT Planning: TOL Working memory (visuospatial): SWMT

CPGI, Canadian Problem Gambling Index; SCID, Structured Clinical Interview for the DSM-IV; IPED, International Personality Disorders Examination; NODS, NORC DSM Screen for Gambling Problems; WASI, Wechsler Abbreviated Scale of Intelligence; WCST, Wisconsin Card Sorting Test; TMT, Trail Making Test; COWAT, Controlled Oral Word Association Test; IGT, Iowa Gambling Task; WMS, Wechsler Memory Scale; SOGS, South Oaks Gambling Screen; WAIS, Wechsler Adult Intelligence Scale; CVLT, California Verbal Learning Test; ROCF, Rey–Osterrieth Complex Figure Test; CFT, Category fluency test; DST, Digit Span Task; RPM, Raven’s Progressive Matrices; BIS, Barratt Impulsiveness Scale; CFQ, Cognitive Failures Questionnaire; DEX, Dysexecutive Questionnaire; SOC, Stockings of Cambridge; IED, Intra-extra Dimensional Set-shift; SWMT, Spatial Working Memory Task; BSI, Brief Symptom Inventory; SST, Stop Signal Task; TOL, Tower of London; SOPT, Self-ordered pointing test; SCI-PG, Structured Clinical Interview for Pathological Gambling; PI-R, Padua Inventory Revised; PRLT, Probabilistic Reversal Learning Task; GSAS, Gambling Symptom Assessment Scale; EIQ, Eysenck Impulsiveness Questionnaire; CGI, Clinical Global Impression-Severity; MIS, Memory Impairment Scale; CIG, Comprehension of Instructions in Greek; SDMT, Symbol Digit Modality Test; CPT, Continuous Performance Task; GRCS, Gambling Related Cognition Scale; MAT, Attentive Matrices; Eqi:S, Emotional Quotient inventory Short; CRT, Frederick’s Cognitive Reflection Test; RSBQ, Frederick’s Risk Seeking Behaviour Task; IBQ, Frederick’s Intemporal Behaviour Task; FDCT, Free Drawn Clock Test; COGST, Cognitive Estimation Test; FLUF, Phonemic and Semantic Verbal Fluency Test; CIDI, Composite International Diagnostic Interview; SSAT, Stop-signal Anticipation Task; UPPS-P: UPPS Impulsive Behaviour Scale; GABS, Gambling Attitudes and Beliefs Survey; GFA, Gambling Functional Assessment; RPP, Retrieval Practice Paradigm; BART, Balloon Analogue Risk Task; DDT, Delayed Discounting Task; CWIT, Color-word Interference Test; G-SAS, Gambling Symptom Assessment Scale; CBTT, Corsi Block-tapping test; MEMINT, Memory of Interference; a, Same sample as Ledgerwood et al., (2012)

Of the 21 studies included in the systematic review, 17 were selected for meta-analysis. Studies were excluded based on the following criteria: (a) Only assessed working memory (*k* = 1)—this domain was not meta-analyzed because there were fewer than three studies assessing it; (b) Insufficient data for meta-analysis (*k* = 1); (c) Same sample used in another included study (*k* = 1); and (d) No statistical association between EF and gambling severity measures was provided (*k* = 1). Due to theoretical restrictions, studies assessing verbal and visuospatial working memory could not be combined in the same analysis.

### Overall Findings

Regarding the shifting domain, the instruments included: Wisconsin Card Sorting Test (WCST; *k* = 7); Trail Making Test (TMT-B; *k* = 5); and Intra-extra Dimensional Set-shift (IED; *k* = 4).

For WCST, most studies (*k* = 4) do not report differences between participants with GD and controls on the number of preservative errors. As for the number of categories completed, two studies (*k* = 2) report that participants with GD complete fewer categories, with one study (Hur et al., 2012) not identifying any differences. For TMT-B, most studies (*k* = 3) report no differences between groups (GD and HC) in the time taken to complete the task. For IED, in the number of errors performed, results are mixed, with one study (Manning et al., 2013) reporting no differences, and another (Choi et al., 2014) reporting a higher number of errors committed by the GD sample. However, both studies show a similar number of stages completed between samples (*k* = 2).

Complementing these results, correlational studies (*k* = 2) show increased shifting deficits at higher gambling severities. Studies not included in the meta-analysis show a negative, non-significant, correlation between GD duration and errors in the IED task (Medeiros et al., 2017). Another study shows the role of depression/dysthymia in performance, with the group with depression/dysthymia performing better than the group with just GD, only on the number of categories completed in the WCST (Ellis et al., 2018).

For the inhibition domain, the instruments included: STROOP (*k* = 8); Stop Signal Task (SST; *k* = 4); Go/No-Go (*k* = 4); Stop-signal Anticipation Task (SSAT; *k* = 2); CPT (*k* = 2); and Color-word Interference Test (CWIT; *k* = 1).

For STROOP, when using the interference score, results are mixed, with one study (Ledgerwood et al., 2012) reporting no differences between groups (GD and HC), and another study (Kapsomenakis et al., 2018) reporting a better performance by controls. When using the time taken to perform the interference task as a metric of performance, no differences are found between groups (*k* = 2). For SST, results are mixed, with some pointing at a higher RT for participants with GD (Kräplin et al., 2014), and others (Choi et al., 2014) reporting no differences. For Go/No-Go, controls have a better performance for false alarms and hit rate (Zhou et al., 2016), while performing less commission errors (Penolazzi et al., 2020). Results related with RT are mixed, with reports for both higher and a similar RT between groups (*k* = 2). For SSAT, two studies (*k* = 2) report a similar RT between groups. For CPT, participants with GD have a higher RT (Kertzman et al., 2018), however when combining this measure with number of hits, no differences are found between participants with GD and HCs (Mestre-Bach et al., 2020b). For CWIT, the reported inhibition score is indicative of a better performance by HC (Aidelbaum et al., 2023).

Studies not included in the meta-analysis show a positive, non-significant, correlation between GD duration and RT in SST (Medeiros et al., 2017). Authors of another study (Leppink et al., 2016b) divided their participants into high and low inhibition groups, as assessed by the SST, with results not showing differences in gambling activity between groups. One study found a correlation between gambling-related cognitions and inhibition, specifically a negative correlation with STROOP errors, and a positive correlation with STROOP completion speed (Schiavella et al., 2018). Depression/dysthymia did not influence inhibition, as assessed by the STROOP interference score (Ellis et al., 2018). One study used the Go/No-Go task without reporting its measures, but results showed no differences between groups (Ledgerwood et al., 2012).

For the planning domain, the instruments included: Tower of London (TOL; *k* = 5); and Stockings of Cambridge (SOC; *k* = 1).

For TOL, a variety of instrument related measures are used to assess task performance. These include rule-breaks (*k* = 3), earned points (*k* = 1), total achievement (*k* = 1) and goal setting (*k* = 1). Specifically for rule-breaks, on one hand, one study (Ledgerwood et al., 2012) reports more rule-breaks by participants with GD, on the other hand, a study (Aidelbaum et al., 2023) reports no differences between groups.

The study not included in the meta-analysis shows that the presence of depression/dysthymia does not influence planning capabilities, as assessed by the number of rule breaks in TOL (Ellis et al., 2018).

For verbal fluency, the instruments included: Controlled Oral Word Association Test (COWAT; *k* = 3); Controlled Oral Word Fluency (COWF; *k* = 1); Phonemic and Semantic Verbal Fluency Test (FLUF/S; *k* = 1); and Category Fluency Test (CFT; *k* = 1). Independent of instrument, most studies (*k* = 5) use the number of correct words as a metric of performance. Reported results (*k* = 3) are not indicative of verbal fluency deficits in participants with GD, excluding one study (Kapsomenakis et al., 2018) that reports a better performance in semantic verbal fluency by the GD sample.

The study not included in the meta-analysis shows that the presence of depression/dysthymia does not influence verbal fluency as assessed by the number of correct words and rule breaks in COWAT (Ellis et al., 2018).

For working memory, the instruments included: Digit Span Task (DST; *k* = 3); Spatial Working Memory Task (SWMT; *k* = 2); Wechsler Memory Scale (WMS; *k* = 2); Self-ordered Pointing Test (SOPT; *k* = 1); Corsi Block-tapping Test (CBTT; *k* = 1); and Memory of Interference (MEMINT; *k* = 1).

For verbal working memory results are inconsistent, as some studies report better performance in the GD sample in the forward DST (Kapsomenakis et al., 2018), while others found a worse performance in this group (Zhou et al., 2016). Similar inconsistency is seen in the backward DST, with results showing either a better performance by the GD sample (Kapsomenakis et al., 2018), or a similar performance to HC (Zhou et al., 2016).

For visuospatial working memory, no differences were found between groups when using the SWMT (*k* = 2). However, for forward and backward CBTT, participants with GD have a better performance (Kapsomenakis et al., 2018).

For overall working memory performance, no differences were found between groups when using the WMS and SOPT (*k* = 2), with the presence of depression/dysthymia not influencing working memory, assessed by the WMS score (Ellis et al., 2018). One study supports a potential association between GD and overall working memory, namely by describing a positive correlation between severity and number of errors MEMINT (Schiavella et al., 2018).

### Meta-Analysis

A description of the included instruments, their measures, the categories they were coded in, and the number of studies and participants included are presented in Table 4.

**Table 4:** Instruments and outcome measures included in the meta-analysis

Construct and instrument	Instrument related measures	Coding	Number of studies (*k*)	*N*
Shifting			10	1082
WCST	Categories completed	Positive	4	395
	Perseverative responses	Negative	5	
	Feedback needed	Negative	1	
TMT-B	Time for completion	RT	4	200
IED	Stages completed	Positive	1	642
	Total errors	Negative	3	
Inhibition			13	1110
STROOP	Interference (Score)	Positive	3	467
	Inhibition index	Negative	1	
	Interference (Time)	RT	3	
SST	Response time	RT	2	134
Go/No-Go	Hit rate	Positive	1	346
	False alarm	Negative	2	
	Commission errors	Negative	1	
	Reaction time	RT	3	
SSAT	Response time	RT	2	138
CPT	False alarm	Negative	1	369
	Commission errors	Negative	1	
	Response time	RT	1	
	Response time (Hit)	RT	1	
CWIT	Inhibition (Score)	Positive	1	90
Planning			5	380
TOL	Earned points	Positive	1	320
	Total achievement	Positive	1	
	Goal setting	Positive	1	
	Rule break	Negative	2	
SOC	Problem solved	Positive	1	60
	Initial thinking time	RT	1	
Verbal fluency			4	307
COWAT (Phonemic)	Total correct	Positive	2	158
	Rule break	Negative	1	
COWF (Phonemic)	Total correct	Positive	1	45
COWF (Semantic)	Total correct	Positive	1	45
FLUF (Phonemic)	Strategy preservation	Negative	1	36
FLUS (Semantic)	Error	Negative	1	36
CFT (Semantic)	Total correct	Positive	1	68

#### Effect Sizes for Shifting

**Fig. 2:**
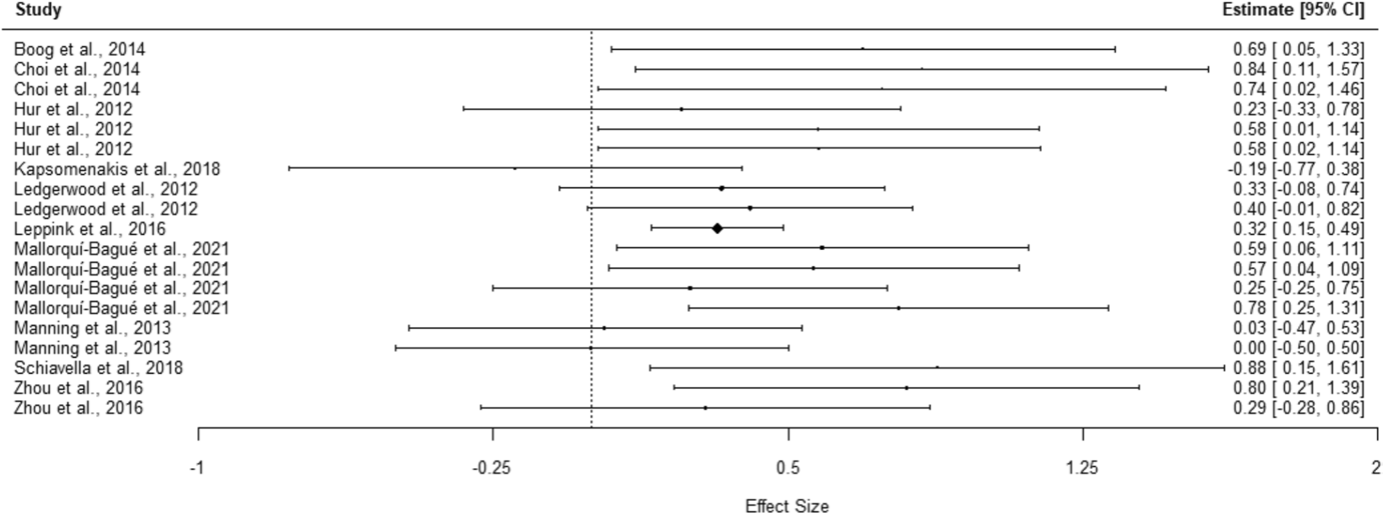
Forest-plot for the shifting meta-analysis

The overall effect size for shifting shows a small, but significant effect of group, with GD performing worse than controls (*g* = 0.40, 95% CI [0.23, 0.57], *p* < 0.001). The heterogeneity test (*Q* = 20.37, *p* = 0.312, *I*^2^ = 11.65%, 95% prediction interval [0.20, 0.60]) did not identify significant variance in results. The *I*^2^ value represents low heterogeneity, supported by a close proximity between the prediction interval and the CI. Meta-analysis results are presented in Table 5, with the corresponding forest plot presented in Fig. 2. Moderator analysis shows that no variable of interest affected the estimated effect size. Two entries were removed as they fulfilled the criteria for outlier deletion.

**Table 5:** Executive function domains and related instruments effect size analysis

Construct, instrument and measures	Studies	*Hedges’ g*	95% *cl*		*SE*	*p*	Heterogeneity test					
									95% prediction values			
			*LL*	*UL*			*Q*	*I*^2^	*LL*	*UL*	*df*	*p*
**Shifting**		0.40	0.23	0.57	0.07	≤ .001	20.37	11.65	0.20	0.60	18	0.312
WCST		0.51	0.35	0.68	0.06	≤ .001	5.92	0	0.49	0.53	10	0.822
Categories completed	Ledgerwood et al., (2012)	0.40	− 0.01	0.82	0.21							
	Hur et al., (2012)	0.58	0.02	1.14	0.29							
	Zhou et al., (2016)	0.29	− 0.28	0.86	0.29							
	Mallorquí-Bagué et al., (2021)	0.78	0.25	1.31	0.27							
Perseveration	Ledgerwood et al., (2012)	0.33	− 0.08	0.74	0.21							
	Hur et al., (2012)	0.58	0.01	1.14	0.29							
	Boog et al., (2014)	0.69	0.05	1.33	0.33							
	Zhou et al., (2016)	0.80	0.21	1.39	0.30							
	Mallorquí-Bagué et al., (2021)	0.25	− 0.25	0.75	0.26							
	Mallorquí-Bagué et al., (2021)	0.57	0.04	1.09	0.27							
Feedback needed	Schiavella et al., (2018)	0.88	0.15	1.61	0.37							
TMT-B		0.32	− 0.32	0.97	0.20	.209	5.44	44.88	− 0.20	0.84	3	0.142
Time	Hur et al., (2012)	0.23	− 0.33	0.78	0.28							
	Choi et al., (2014)	0.74	0.02	1.46	0.37							
	Kapsomenakis et al., (2018)	− 0.19	− 0.77	0.38	0.29							
	Mallorquí-Bagué et al., (2021)	0.59	0.06	1.11	0.27							
IED		0.29	− 0.52	1.10	0.14	.214	4.61	34.96	0.01	0.57	3	0.202
Stages completed	Manning et al., (2013)	0.00	− 0.50	0.50	0.25							
Total errors	Manning et al., (2013)	0.03	− 0.47	0.53	0.25							
	Choi et al., (2014)	0.84	0.11	1.57	0.37							
	Leppink et al., (2016a)	0.32	0.15	0.49	0.09							
**Inhibition**		0.34	0.28	0.41	0.02	≤ .001	39.02	43.61	− 0.05	0.73	22	0.014
STROOP		0.31	0.01	0.62	0.10	.047	5.00	0	0.29	0.33	6	0.543
Interference (Score)	Ledgerwood et al., (2012)	0.27	− 0.14	0.68	0.21							
	Kapsomenakis et al., (2018)	0.72	0.12	1.31	0.30							
	Mallorquí-Bagué et al., (2021)	0.11	− 4.10	4.31	2.15							
Inhibition index	Albein-Urios et al., (2012)	0.67	0.07	1.28	0.31							
Interference (Time)	Mallorquí-Bagué et al., (2021)	0.11	-5.05	5.27	2.63							
	Hur et al., (2012)	-0.03	-0.59	0.52	0.28							
	Kräplin et al., (2014)	0.21	-0.17	0.59	0.20							
SST												
Reaction time	Choi et al., (2014)	0.01	-0.69	0.70	0.36							
	Kräplin et al., (2014)	0.47	0.08	0.86	0.20							
Go/No-go		0.41	0.03	0.79	0.06	.045	19.80	69.70	-0.21	1.03	6	0.003
Hit rate	Zhou et al., (2016)	0.67	0.09	1.26	0.30							
False alarm	Zhou et al., (2016)	0.66	0.07	1.24	0.30							
	Kertzman et al., (2018)	0.18	-0.07	0.43	0.13							
Commission errors	Penolazzi et al., (2020)	0.64	0.13	1.16	0.26							
Reaction time	Zhou et al., (2016)	0.02	− 0.55	0.58	0.29							
	Kertzman et al., (2018)	0.74	0.48	1.00	0.13							
	Penolazzi et al., (2020)	− 0.24	− 0.74	0.27	0.26							
SSAT												
Reaction time	Sharif-Razi et al., (2019)	0.30	− 0.27	0.86	0.29							
	Aidelbaum et al., (2023)	0.44	− 0.09	0.98	0.27							
CPT												
False alarm	Kertzman et al., (2018)	0.02	− 0.23	0.27	0.13							
Commission errors	Mestre-Bach et al., (2020b)	0.60	0.19	1.00	0.21							
Reaction time	Kertzman et al., (2018)	0.49	0.24	0.75	0.13							
Reaction time (Hit)	Mestre-Bach et al., (2020b)	0.01	− 0.38	0.41	0.20							
CWIT												
Inhibition (Score)	Aidelbaum et al., (2023)	0.45	0.01	0.89	0.23							
**Planning**		0.06	− 0.45	0.58	0.18	.755	14.31	58.08	− 0.49	0.61	6	0.026
TOL		0.15	− 0.58	0.89	0.23	.545	12.56	68.16	− 0.50	0.80	4	0.014
Earned points	Kräplin et al., (2014)	− 0.17	− 0.55	0.21	0.20							
Total achievement	Aidelbaum et al., (2023)	− 0.14	− 0.56	0.29	0.22							
Goal setting	Schiavella et al., (2018)	0.73	0.02	1.44	0.36							
Rule break	Ledgerwood et al., (2012)	0.50	0.09	0.92	0.21							
	Aidelbaum et al., (2023)	− 0.30	− 0.73	0.13	0.22							
SOC												
Problem solved	Manning et al., (2013)	− 0.21	− 0.71	0.29	0.26							
Initial thinking time	Manning et al., (2013)	− 0.27	− 0.77	0.23	0.26							
**Verbal fluency**		0.14	− 0.65	0.93	0.25	.612	20.07	65.12	− 0.60	0.87	7	0.005
COWAT												
Phonemic—total correct	Ledgerwood et al., (2012)	0.14	− 0.27	0.55	0.21							
	Hur et al., (2012)	0.45	− 0.11	1.01	0.29							
Phonemic—rule break	Ledgerwood et al., (2012)	0.19	− 0.22	0.60	0.21							
COWF												
Phonemic—total correct	Kapsomenakis et al., (2018)	− 0.25	− 0.82	0.33	0.29							
Semantic—total correct	Kapsomenakis et al., (2018)	− 0.63	− 1.22	− 0.04	0.30							
FLUF/S												
Phonemic—perseveration	Schiavella et al., (2018)	0.71	− 0.00	1.42	0.36							
Semantic—error	Schiavella et al., (2018)	1.01	0.26	1.76	0.38							
CFT												
Semantic—total correct	Hur et al., (2012)	− 0.36	− 0.92	0.20	0.28

The overall effect size for WCST (*g* = 0.51, 95% CI [0.35, 0.68], *p* < 0.001) shows a moderate, significant effect of group, with GD performing worse. The heterogeneity statistics (*Q* = 5.92, *p* = 0.822, *I*^2^ = 0%, 95% prediction interval [0.49, 0.53]) did not identify significant variance in results. One entry was removed as it fulfilled the criteria for outlier deletion.

The overall effect size for TMT-B (*g* = 0.32, 95% CI [− 0.32, 0.97], *p* = 0.209) shows a small and non-significant effect of group, even if GD shows worse performance. The heterogeneity test (*Q* = 5.44, *p* = 0.142, *I*^2^ = 44.88%, 95% prediction interval [− 0.20, 0.84]) did not identify significant variance in results; however the *I*^2^ value represents moderate heterogeneity, with a wide prediction interval supporting these findings. One entry was removed as it fulfilled the criteria for outlier deletion.

The overall effect size for IED (*g* = 0.29, 95% CI [− 0.52, 1.10], *p* = 0.214) shows a small and non-significant effect of group, even if GD shows worse performance. The heterogeneity test (*Q* = 4.61, *p* = 0.202, *I*^2^ = 34.96%, 95% prediction interval [0.01, 0.57]) did not identify significant variance in results; however the *I*^2^ value represents low to moderate heterogeneity, with the difference between the prediction interval and the CI supporting these findings.

#### Effect Sizes for Inhibition

**Fig. 3:**
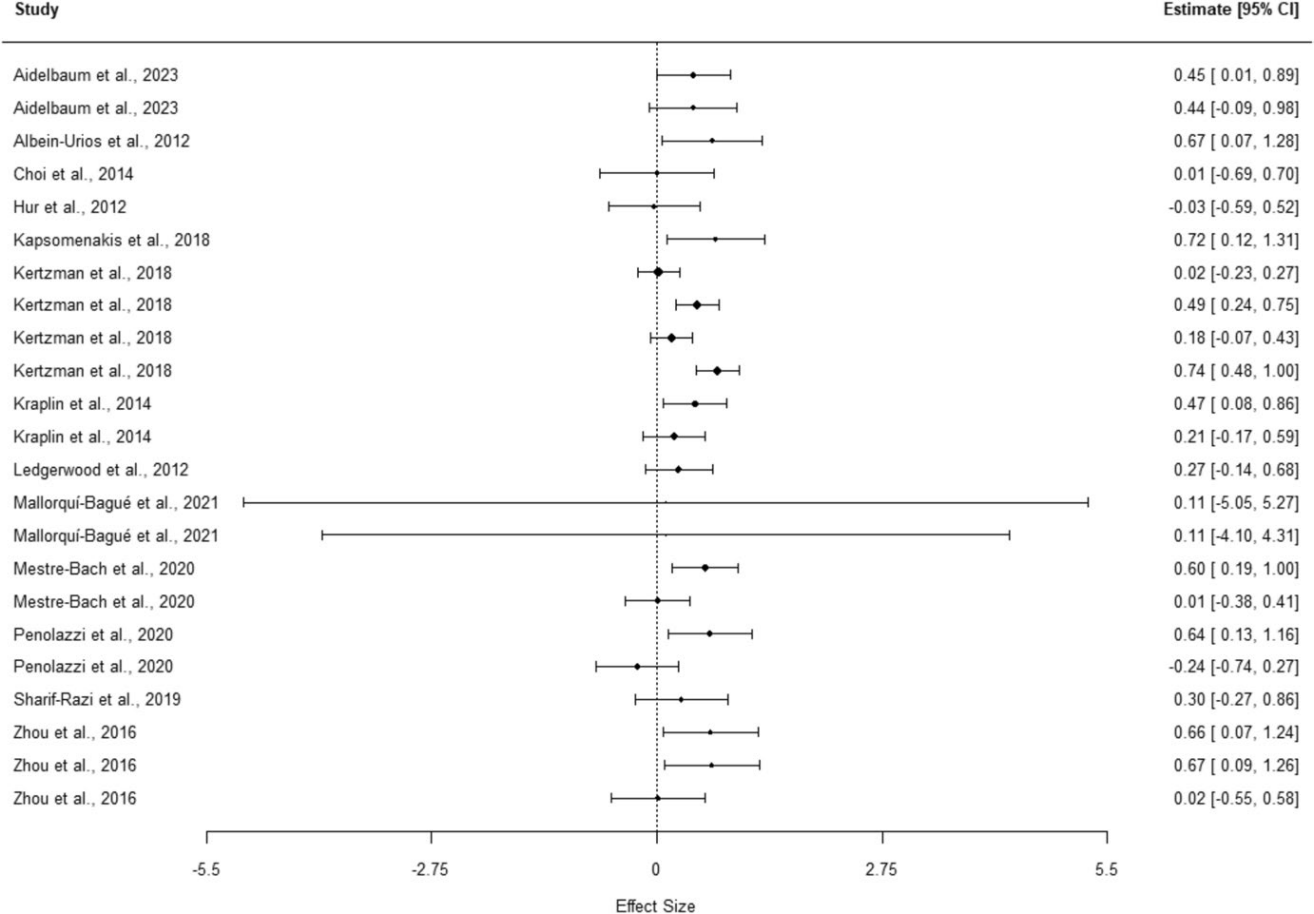
Forest-plot for the inhibition meta-analysis

The overall effect size for inhibition shows a small, but significant effect of group, with GD performing worse than controls (*g* = 0.34, 95% CI [0.28, 0.41], *p* ≤ 0.001). The heterogeneity test (*Q* = 39.02, *p* = 0.014, *I*^2^ = 43.61%, 95% prediction interval [− 0.05, 0.73]) identified significant variance in results. The *I*^2^ value represents moderate heterogeneity, with a wide prediction interval and its difference from the CI supporting these findings. Meta-analysis results are presented in Table 5, with the corresponding forest plot in Fig. 3. Moderator analysis shows that no variable of interest affected the estimated effect size.

The overall effect size for STROOP (*g* = 0.31, 95% CI [0.01, 0.62], *p* = 0.047) shows a small, but significant effect of group, with GD performing worse. The heterogeneity test (*Q* = 5.00, *p* = 0.543, *I*^2^ = 0%, 95% prediction interval [0.29, 0.33]) did not identify significant variance in results.

The overall effect size for Go/No-Go task (*g* = 0.41, 95% CI [0.03, 0.79], *p* = 0.045) shows a small, but significant effect of group, with GD performing worse. The heterogeneity test (*Q* = 19.80, *p* = 0.003, *I*^2^ = 69.70%, 95% prediction interval [-0.21, 1.03]) identified significant variance in results. The *I*^2^ value represents substantial heterogeneity, with a wide prediction interval and its difference from the CI supporting these findings. Other reported instruments do not meet the criteria, as they were reported in fewer than three studies.

#### Effect Sizes for Planning

**Fig. 4:**
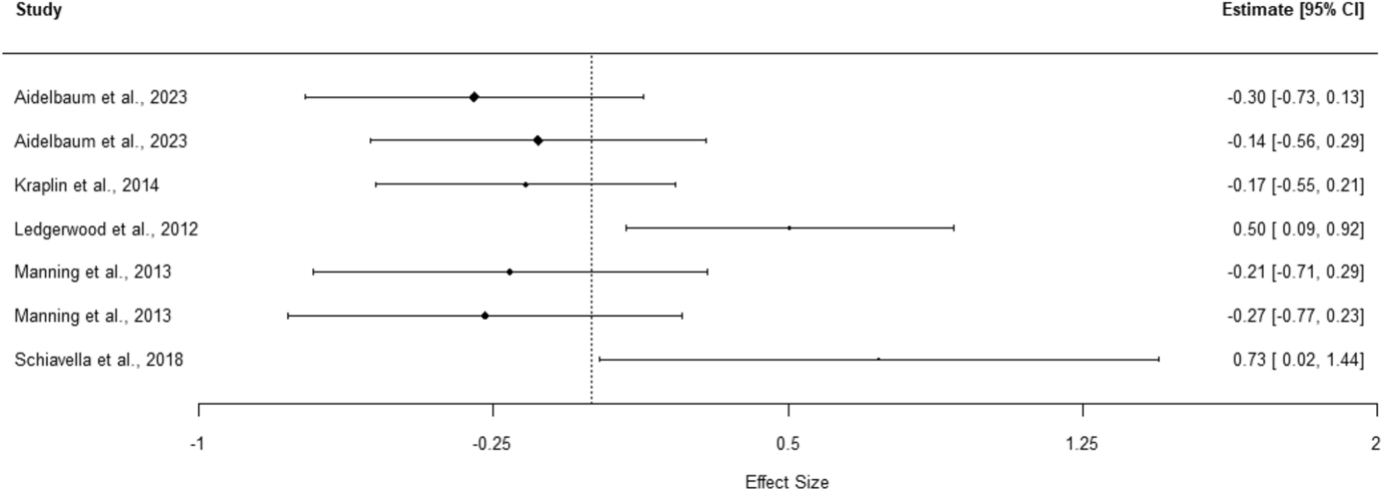
Forest-plot for the planning meta-analysis

The overall effect size for planning shows a negligible and non-significant effect of group (*g* = 0.06, 95% CI [− 0.45, 0.58], *p* = 0.755). The heterogeneity test (*Q* = 14.31, *p* = 0.026*, I*^2^ = 58.08%, 95% prediction interval [− 0.49, 0.61]) identified significant variance in results. The *I*^2^ value represents substantial heterogeneity, with a wide prediction interval supporting these findings. Meta-analysis results are presented in Table 5, with the corresponding forest plot presented in Fig. 4. Moderator analysis shows that no variable of interest affected the estimated effect size.

The overall effect size for TOL (*g* = 0.15, 95% CI [-0.58, 0.89], *p* = 0.545) shows a negligible and non-significant effect of group, even if GD shows worse performance. The heterogeneity test (*Q* = 12.56, *p* = 0.014, *I*^2^ = 68.16%, 95% prediction interval [-0.50, 0.80]) identified significant variance in results. The *I*^2^ value represents substantial heterogeneity, with a wide prediction interval supporting these findings. Other reported instruments do not meet the criteria, as they were reported in fewer than three studies.

#### Effect Sizes for Verbal Fluency

**Fig. 5:**
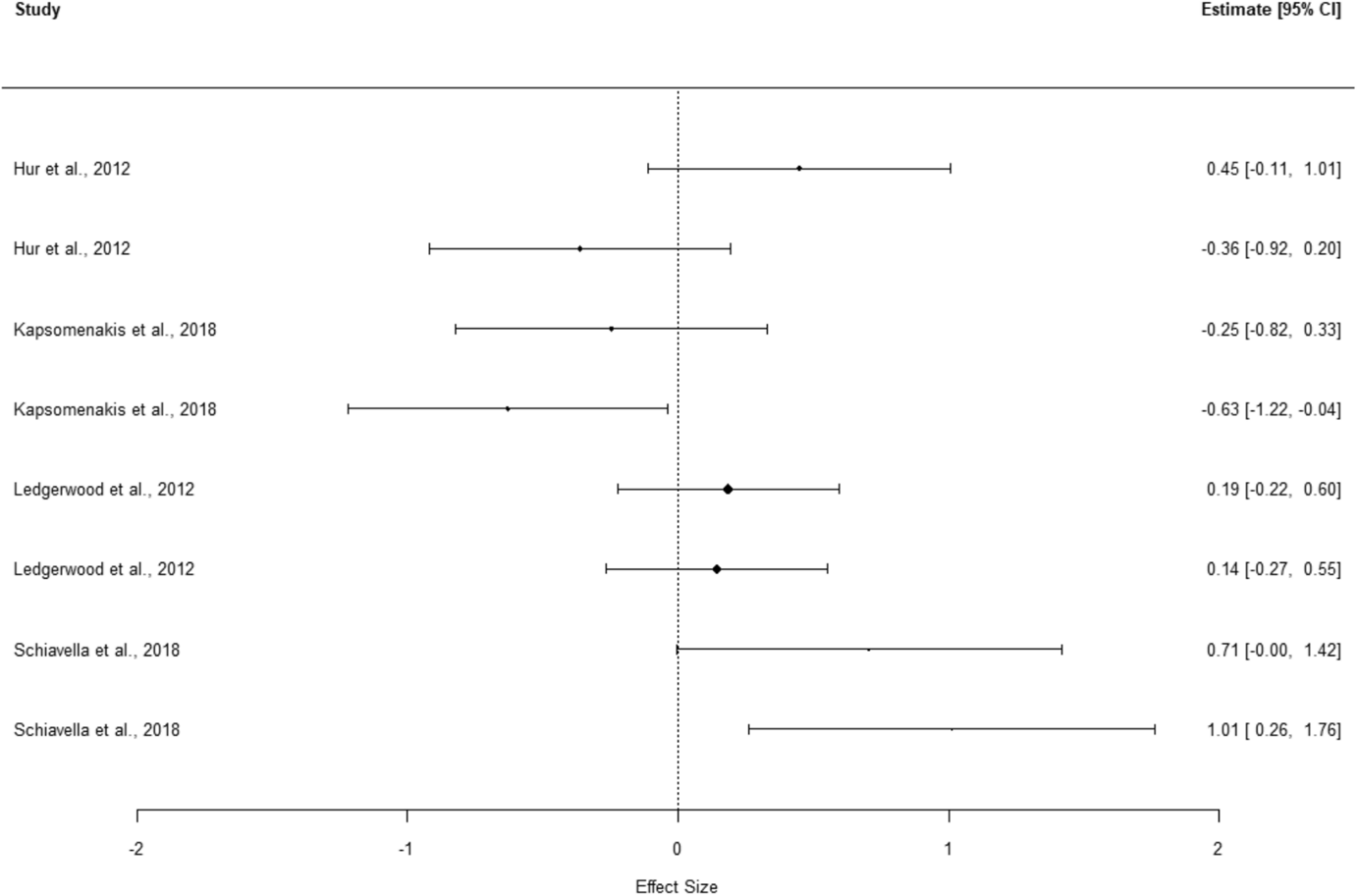
Forest-plot for the verbal fluency meta-analysis

The overall effect size for verbal fluency shows a negligible and non-significant effect of group (*g* = 0.14, 95% CI [− 0.65, 0.93], *p* = 0.612), even if GD shows a worst performance. The heterogeneity test (*Q* = 20.07, *p* = 0.005, *I*^2^ = 65.12%, 95% prediction interval [− 0.60, 0.87]) identified significant variance in results. The *I*^2^ value represents substantial heterogeneity, with a wide prediction interval supporting these findings. Meta-analysis results are presented in Table 5, with the corresponding forest plot presented in Fig. 5. Moderator analysis shows that no variable of interest affected the calculated effect size.

### Publication bias

The results of the publication bias analysis are presented in Table 6. The shifting domain had four studies trimmed through the trim-and-fill method, with the effect size remaining significant. The inhibition domain had four studies trimmed through the trim-and-fill method, with the effect size remaining significant. The verbal fluency domain had one study trimmed through the trim-and-fill method.

**Table 6:** Publication bias analysis

Executive function	Hedge’s g—observed	Effect size—estimated (trimmed studies)	Egger’s Test
			Interception (*p*)
Shifting	0.40	0.33 (4)	0.86 (0.138)
Inhibition	0.34	0.42 (4)	0.04 (0.995)
Planning	0.06	0.06 (0)	3.06 (0.427)
Verbal fluency	0.14	0.03 (1)	1.72 (0.593)

## Discussion

This study aimed to examine EF deficits in participants with GD, using standardized neuropsychological instruments that assess the main executive functioning domains, based on literature published after 2012. Given the link between EF and addictive behaviors (Kräplin et al., 2022), reviewing and confirming these deficits is an important step to further our understanding of GD. With a previous review on the topic being met with contradictory evidence (Conversano et al., 2012), an updated systematic review and a meta-analysis was deemed the appropriate method to examine the current evidence on the subject and more accurately inform future research. With this goal in mind, the meta-analysis was intended to test the following hypothesis: Shifting, inhibition, and planning are significantly impaired in participants with GD, while verbal fluency and working memory are not. In addition to the goal above, this review also identifies and describes the instruments commonly used to assess the main domains of EF, helping to determine the source of the measures that distinguish the executive functioning of individuals with GD from HC.

Initial data review relating to the shifting domain seems to indicate an absence of shifting deficits in participants with GD, especially when considering TMT results (Choi et al., 2014; Hur et al., 2012; Kapsomenakis et al., 2018). In contradiction, the number of categories formed in WCST appears as a more consensual measure in identifying shifting deficits in samples of gamblers (Ledgerwood et al., 2012; Zhou et al., 2016). Complementing this data, for IED, one set of results is indicative of shifting deficits in participants with GD, based on the number of errors (Choi et al., 2014). Thus, varying degrees of evidence are found, which is expected as Conversano et al. (2012) found similar data. In addition, correlational studies (Leppink et al., 2016a; Schiavella et al., 2018) show a negative correlation between pathology severity and performance in shifting tasks. Indeed cognitive inflexibility is shown to be a characteristic of behavioral addictions (Lim & Ersche, 2024), and shifting is considered a compulsivity-related neurocognition (Van Timmeren et al., 2018), a core construct in behavioral addictions (Lee et al., 2019). Thus, despite a qualitative analysis revealing inconsistent data, there seems to be a trend towards a worst performance in shifting tasks by GD samples. Comorbid symptomatology could potentially influence results. Interestingly, a GD sample with depression/dysthymia performed better than a sample without depression/dysthymia, on the number of categories completed in the WCST (Ellis et al., 2018). On the contrary, anxiety seems to negatively influence performance efficiency on the WCST in healthy samples (Edwards et al., 2015). Thus, varying degrees of this pathology across samples of GD participants can potentially contribute towards the observed heterogeneity in results.

For the inhibition domain, results are mixed, even when considering each instrument separately. Despite this, results vary between no differences between groups and a worst performance in the GD sample. Stroop interference score (Kapsomenakis et al., 2018), SST RT (Kräplin et al., 2014), CPT RT (Kertzman et al., 2018) and errors (Mestre-Bach et al., 2020b), and Go/No-Go RT (Kertzman et al., 2018), false alarms (Zhou et al., 2016), hit rate (Zhou et al., 2016) and commission errors (Penolazzi et al., 2020), appear as indicators of poorer inhibition in GD samples. This set of results are expected as gamblers’ behavior is characterized by high impulsivity (Ioannidis et al., 2019), possibly resulting in a harmful, continued gambling activity (Devos et al., 2015). Complementing these findings, one study showed a negative correlation between gambling-related cognitions and inhibition, but did not found a significant correlation between PGSI and SOGS scores (Schiavella et al., 2018). Previous results have shown a positive correlation between impulsivity driven by an emotional state and/or urgency and gambling-related cognitions (Del Prete et al., 2017; Marmurek et al., 2015). Thus, the association found between gambling-related cognitions and poorer performance in the inhibition task could be caused by the participants emotional state, and not due to a lack of cognitive capacity to inhibit a prominent response pattern. Nonetheless, and contrary to shifting, depression/dysthymia does not influence performance in inhibition tasks (Ellis et al., 2018), regardless of other studies establishing a positive correlation between impulsivity and depression (Clarke, 2006). Contradictory results could be explained by varying degrees in depression severity, as studies show a worst performance on the STROOP task in more severe cases, compared to less severe ones (Markelalerenc et al., 2006; Medeiros et al., 2014). These results point toward the confirmation of inhibition deficits in GD participants.

For the planning domain, only one study (Ledgerwood et al., 2012) identified a difference between groups, with the GD sample having a worst performance, assessed through number of rule-breaks in TOL. Given that lack of premeditation is a key facet of impulsivity (Cyders et al., 2014), it would be expected that more studies would find deficits in planning capacity. The differences in results could be caused by varying degrees of gambling severity of the included samples, as proposed by Conversano et al. (2012). The presence of depression/dysthymia does not influence planning capabilities, as assessed by the number of rule breaks in TOL (Ellis et al., 2018). This is to be expected, as the difference between people with depression and HC is the time that it takes to perform the planning task, and not in the level of their performance (Goethals et al., 2005), as assessed by the number of rule-breaks.

For verbal fluency, only one study (Kapsomenakis et al., 2018) identified differences between groups, namely that GD participants have a better performance in COWF. Evidence on verbal fluency in GD has been mixed (Goudriaan et al., 2006; Marazziti et al., 2008), which is also noted in studies on internet addiction (Choi et al., 2014; Nie et al., 2017). Depression/dysthymia does not influence verbal fluency (Ellis et al., 2018), which would be expected given that participants’ mood seems to influence phonemic fluency (Clark et al., 2001), although there is data that suggests that verbal fluency deficits in depression varies across samples (Klumpp & Deldin, 2010). These results are indicative of no verbal fluency deficits in GD participants.

Results for working memory are varied, with some indicating deficits in participants with GD (Zhou et al., 2016), others showing a better performance (Kapsomenakis et al., 2018), and others showing no deficits (Zhou et al., 2016). The results remain inconsistent when analyzing visuospatial and verbal working memory separately. A recent systematic review indicates an association between GD and working memory, with deficits appearing to be task-dependent (Ngetich et al., 2023), similar to our current analysis. As with other EF domains, anxiety seems to be associated with worse working memory performance (Moran, 2016). Neuroimaging data indicates that a crucial brain structure for working memory, the dorsolateral prefrontal cortex (dlPFC; Barbey et al., 2013), is hypoactivated in participants with GD (Raimo et al., 2021), corroborating evidence of working memory deficits in this population. The results are in line with a high heterogeneity of results in this EF domain, which hinders the interpretation of potential deficits in working memory in participants with GD. Thus, more studies are necessary to confirm working memory deficits in GD samples. Related to these findings, there is a need for more studies employing tasks that more accurately assess the updating domain, as neuroimaging data shows that certain brain structures involved in updating are not involved in other aspects of working memory, namely maintenance (Trutti et al., 2021). Studies that assess updating through N-back show that the performance of individuals with substance addiction (e.g., methamphetamine, alcohol) is indicative of deficits in this domain (Firoozabadi et al., 2023; Gupta et al., 2018), thus meriting further investigation of this domain in the context of GD, given the neural substrates common to both addictions (Potenza, 2006; Verdejo-Garcia et al., 2015).

Regarding the results of the meta-analysis, the shifting domain is impaired in participants with GD, which aligns with previous results (Van Timmeren et al., 2018). Despite this, a previous review highlights inconsistencies at this level (Conversano et al., 2012). Heterogeneity in instruments and related measures could explain some of the difficulties in mapping precise deficits in shifting. The various degrees of gambling severity encompassed under the umbrella term of GD, can also explain the variety of results, as deficits are more prominent at higher severity levels (Odlaug et al., 2011), with a higher percentage of shifting-related errors occurring at greater gambling severities (Leppink et al., 2016a). Despite varying degrees of evidence, heterogeneity is not significant, suggesting that shifting deficits are present in most GD samples, also identified in self-report measures (Reid et al., 2012). Furthermore, the prediction interval shows a high likelihood that future studies find a positive effect size indicative of impairments in the shifting domain in participants with GD. These results are supported by neuroimaging data showing that gamblers have reduced white matter between the dlPFC and basal ganglia, an essential circuit for cognitive flexibility (Van Timmeren et al., 2017). More in-depth characterization of variables related to gambling severity could potentially explain the studies that do not detect deficits in shifting. These results support the hypothesis of shifting deficits in participants with GD.

Regarding the instruments used to assess shifting, the WCST is the most homogeneous instrument for assessing EF deficits in participants with GD. This is contrary to previous reports (Conversano et al., 2012), although recent evidence confirms the current findings (Quintero, 2016). Unlike the WCST, IED results do not support the ability of this instrument to identify shifting deficits in GD, despite the effect size suggesting such deficits. The limited number of studies using this instrument or the dependency between effect sizes may explain these findings. The TMT-B, which previously showed significant differences between samples (Van Timmeren et al., 2018), did not reveal such differences in this study. Higher heterogeneity in TMT-B results could explain the difference between studies. This heterogeneity might arise from using RT instead of the difference between TMT-A and TMT-B as a measure of shifting, which would lower the impact of visuoperceptual and motor demands on the results (Sánchez-Cubillo et al., 2009).

The effect size for the inhibition domain, revealed a significant deficit in GD samples. As previously stated, given that gamblers’ behavior is characterized as impulsive (Ioannidis et al., 2019), these results were expected. Neuroimaging data corroborates these findings, showing decreased responsiveness in the dorsomedial prefrontal cortex (dmPFC) during successful response inhibition and in the anterior cingulate cortex (ACC) during unsuccessful response inhibition (Moccia et al., 2017), areas associated with cognitive control (Van Holst et al., 2012). Despite promising evidence, interpretations should be made with caution due to a significant impact of heterogeneity on effect sizes. This heterogeneity may arise from measures collected in different instruments, as the SST and Go/No-Go task, which have been associated with considerable variability (Chowdhury et al., 2017). The potential presence of uncharacterized psychopathology in the included samples, could also contribute to differing results, as certain pathologies are known to affect motor impulsivity (Wright et al., 2014). The personality profile of the included samples could also explain some of the heterogeneity, as data indicates a potential association between gambling and impulsivity as a stable trait of the individual (Lai et al., 2011). Hence, the results support the hypothesis that inhibition is significantly impaired in participants with GD, but significant heterogeneity may be influencing the effect sizes.

As for the instruments used to assess inhibition, the STROOP appears to be the more consensual instrument in detecting deficits in participants with GD. Contrary to previous results, no heterogeneity in effect sizes was found (Van Timmeren et al., 2018). Despite high heterogeneity, Go/No-Go task results also support its capability in identifying inhibition deficits in GD, as found in the literature (Kertzman et al., 2008). The high heterogeneity could be attributed to the use of time as a metric of performance, which when interpreted without accuracy is more susceptible to motor impulsivity. For other instruments, a more in-depth analysis was not possible; however, effect size values suggest a trend towards worse performance by participants with GD. This data aligns with previous findings showing overall poorer performance by participants with GD in SST (Chowdhury et al., 2017).

The effect size for the planning domain, revealed no significant deficits in individuals with GD and high heterogeneity. Previous research shows deficits in planning (Goudriaan et al., 2006), being cited as the most consistently observed EF deficit (Ledgerwood et al., 2012). Our current results could be partly explained by the inclusion of a wider range of TOL-related measures, which may provide a more accurate assessment of performance compared to using a single measure (Berg et al., 2010). Neuroimaging data also corroborates the idea that planning is intact in individuals with GD, showing preserved dorsal frontostriatal circuit activation (De Ruiter et al., 2009). Additionally, comorbidities such as anxiety can influence planning performance (Unterrainer et al., 2018), potentially explaining the varying degrees of evidence. Thus, results do not support the hypothesis of planning deficits in participants with GD. However, the high heterogeneity in effect sizes and a small sample of studies limit this claim.

Regarding the assessment of planning, two instruments were used, with TOL being the most frequently reported. Results on TOL are not indicative of significant deficits in participants with GD. Interpretation of these results should be made carefully due to high heterogeneity and small sample sizes. In turn, high heterogeneity could be explained by varying degrees of gambling severity, as proposed by Conversano et al. (2012). Other variables such as differences in inhibitory control (Luciana et al., 2009), and even cultural differences (Phillips et al., 2021) could impact performance on TOL, resulting in high heterogeneity.

The effect size for verbal fluency, revealed no significant deficits in GD samples and significant heterogeneity. Verbal fluency is assessed by a variety of instruments, including both phonemic and semantic modalities. As mentioned in the qualitative analysis, few studies assess verbal fluency in GD, and results are inconsistent (Goudriaan et al., 2006; Marazziti et al., 2008). The influence of participant’s mood on phonemic verbal fluency could explain the variability in results (Clark et al., 2001). In addition, combining phonemic and semantic verbal fluency could affect the calculated effect size, as semantic verbal fluency has a stronger association with other EF domains (Aita et al., 2019). These results confirm our initial hypothesis that verbal fluency is not impaired in participants with GD. However, the high heterogeneity in effect sizes and a small sample of studies limit this claim.

Complementary to these analyses, sample-related data was also gathered to characterize and assess potential variables that could explain the EF profile in GD. Age, gender, and years of formal education were considered, with none of these variables explaining the results, which was expected based on previous analyses (Van Timmeren et al., 2018). Most of the samples in such analyses were in treatment and, when compared to gamblers recruited from the community, show higher impulsivity (Knezevic & Ledgerwood, 2012) and lower performance in shifting and verbal fluency tasks (Ledgerwood et al., 2012).

Conclusions drawn from the data analyzed in the current review should consider study limitations. The first limitation is related to the search string used, given the variability in nomenclature for gambling-related variables, which could have led to missing studies that would fit the inclusion criteria. The second limitation is the exclusion of gambling severity and treatment status from the moderator analysis, but such analysis could not be performed due to a lack of data. Given the limitations on the diagnostic accuracy of the instruments used to assess gambling severity (Williams & Volberg, 2014), samples’ scores are necessary to more accurately understand and interpret the reported results. Additionally, the lack of data on important comorbidities limits the generalizability of results. The third limitation is that the theoretical model used to organize EF domains does not encompass other suggested domains, such as decision-making. As such, conclusions regarding other potential EF domains remain open.

As for future recommendations, studies should consider reporting comorbidities that may influence task performance, especially depression and anxiety, given their prevalence in GD samples. Portraying to gambling severity, a strong recommendation is to report the average score obtained by the sample in measures of gambling behavior (i.e. SOGS, PGSI). The first reason for this recommendation is the fact that an umbrella term like “GD” or “problematic gambler” encompasses varying degrees of gambling severity; thus, additional information on gambling severity would contribute towards a better understanding regarding the variability in results. The second reason for this is the inherent limitations of instruments such as the SOGS and the PGSI, which can lead to difficulties in accurately categorizing the participant. Tied to these points, is the recommendation for more studies to approach gambling disorder as a continuous variable, potentially enabling a more detailed look into EF deficits presented in the gambling community. Of note, more homogeneity amongst studies in the instruments and the related measures used would facilitate result comparison. Finaly, it is worth mentioning that the use of time as a measure of EF performance may not be accurate, as motor response impulsivity can interfere with the results. This is especially relevant for studies that interpret RT independently from accuracy.

## Conclusion

Altogether, the reviewed studies seem to provide sufficient evidence of deficits in shifting and inhibition in samples with GD. However, results do not support a deficit in planning and verbal fluency. High variability in instruments used and in instrument-related measures could explain why results do not show a consistent pattern across the literature. Thus, considering a theoretical framework that incorporates relevant EF domains and the instruments and related measures used for their assessment, could provide a consistent basis for neuropsychological assessment in GD across studies.

The lack of necessary data for sample characterization is another hindrance to drawing broader conclusions, as relevant information is missing from studies. This issue is especially relevant when categorizing the samples as problematic gamblers, pathological gamblers, or having GD. The limitations inherent in the instruments used to assess severity, along with the variability in gambling-related nomenclature across studies, are significant barriers to understanding EF deficits across different degrees of gambling severity. Therefore, more emphasis should be given on reporting scores obtained from gambling severity scales. An additional approach is to consider gambling severity as a continuous variable rather than categorical.

Gambling disorder is a complex behavioral addiction encompassing numerous risk and maintenance factors. In the context of EF, more studies should adopt a longitudinal approach to better understand whether EF deficits exist before disorder development, if they are a consequence of it, or if both etiological paths are possible, as some theoretical models propose. Empirical validation of theoretical models is a necessary step in gambling research, to standardize study methodologies and consolidate the understanding of the mechanisms underlying GD formation and maintenance.

In summary, despite inconsistencies, GD seems selectively associated with deficits in certain EF domains, as revealed by poorer performance of samples with GD in tasks that assess shifting and inhibition. Given the importance of these functions in the development and maintenance of GD, as well as in activities of daily living, clarifying these deficits is fundamental for developing neurocognitive rehabilitation programs that effectively address the most prevalent deficits in GD.

## Data Availability

Data will be made available upon request. The pre-registration protocol is available in PROSPERO: ID CRD42024569344. Hypotheses identified in the manuscript were not pre-registered.
